# Rapid Determination of 226Ra and Uranium Isotopes in Solid Samples by Fusion with Lithium Metaborate and Alpha Spectrometry

**DOI:** 10.1100/tsw.2002.821

**Published:** 2002-07-09

**Authors:** R. Bojanowski, Z. Radecki, R. Piekoś

**Affiliations:** Institute of Oceanology, PAS, ul. Powstanców Warszawy 55, 81-712 Sopot, Poland; International Atomic Energy Agency, Seibersdorf Laboratories, P.O. Box 100, A-1400 Vienna, Austria; Medical University of Gdansk, Faculty of Pharmacy, Al. Gen. Hallera 107, 80-416 Gdansk, Poland

**Keywords:** radium-226, uranium isotopes, determination, alpha-spectrometry, rapid radiochemical methods

## Abstract

A simple and rapid method has been developed to determine ^226^Ra in rocks, soils, and sediments. Samples are decomposed by fusion with lithium metaborate and the melt is dissolved in a solution containing sulfates and citric acid. During the dissolution, a fine suspension of mixed barium and radium sulfates is formed. The microcrystals are collected on a membrane filter (pore size 0.1 μm) and analysed in an alpha spectrometer. Application of a ^133^Ba tracer enables us to assess the loss of the analyte, which only rarely exceeds 10%. All analytical operations, beginning from sample decomposition to source preparation for alpha spectrometry, can be accomplished within 1 or 2 h.

With uranium determination, the filtrate is spiked with a ^232^U tracer and passed through a column loaded with a Dowex AG (1 x 4) anion-exchange resin in the sulfate form. Interfering elements are eluted with dilute sulfuric acid followed by concentrated hydrochloric acid. Uranium is eluted with water, electrodeposited on silver discs, and analysed in the alpha spectrometer. The method was tested on reference soil and sediment materials and was found to be accurate within the estimated uncertainties.

### INTRODUCTION

$^{226}$Ra is the earliest and one of the most frequently measured natural radionuclides. Hundreds of methods, and their modifications, have been devised and developed since its discovery by the eminent Polish scientist and Nobel Prize winner, Mme. Maria Sklodowska-Curie in 1898[1]. These differ widely in many respects, but all of them can be classified into one of the two following categories: those which are based on a specific property of the $^{226}$Ra as such, and those which depend on the properties of its decay products $^{222}$Rn, $^{218}$Po, $^{214}$Pb, and $^{214}$Bi. The latter are the most popular because they offer either high sensitivity and specificity (radon emanation) or require little effort for sample preparation and measurement. However, they are not fast, because time is consumed when samples lay in wait for the decay products to build up. Usually, the samples are kept in gas-proof vessels for at least 2 weeks or more during which the secular equilibrium within the decay chain is approached. It follows from the foregoing, that the time of analysis can be reduced considerably when $^{226}$Ra is measured directly.

Among the methods that offer such a possibility are those which are based on counting atoms (ICP-MS, TIMS, AMS) and those which count alpha particles and/or gamma photons generated by the decaying $^{226}$Ra nuclei. Methods exploiting gamma radiation at 186.1 keV, while interesting for their simplicity, have limited application because of low sensitivity (line intensity of 3.53%) and strong interference from the $^{235}$U (I = 57.2% at E = 185.7 keV). All remaining methods require extensive sample processing which includes dissolution of the matrix, removal of interfering elements, and preparation of the sample in a form suitable for final determination. Each step is more or less time consuming and labor intensive, and it is there where the potential for improvements exists.

When the final determination of $^{226}$Ra is performed by alpha spectrometry, the source should be weightless to prevent the spectrum from degradation due to peak broadening. It should also be free of the radionuclides that emit alpha particles in the $^{226}$Ra energy region ($^{234}$U, $^{230}$Th). Solid samples must be totally decomposed to avoid uncontrolled loss of the analyte to the insoluble particles. The most popular approach is dissolution in a mixture of HF and mineral acid such as HCl, $HNO_3$, $HClO_4$ or $H_2SO_4$. These methods are, however, lengthy and do not ensure complete dissolution of all components of the analysed material. Some mineral components of soils and sediments such as zircon, topaz, corundum, sillimanite, tourmaline, rutile, and barite are known to resist attack with acids unless prolonged heating under high pressure is applied[2]. More radical decomposition methods include fusion with hydroxides, peroxides, carbonates, fluorides, pyrosulfates, and borates of alkaline elements.

The first three fluxes require subsequent removal of silica and separation of radium from the bulk salts and interfering elements. These operations are tedious, hence unattractive. Sill[3] described a method in which samples are decomposed by fusion with potassium fluoride followed by evaporation with sulfuric acid and melting of the residue with sodium pyrosulfate. This procedure offers considerable advantage in that the dissolution of sample is complete, no matter how refractory the material is, and that no complex separation schemes are needed to prepare radium sources for alpha spectrometry. It is, however, inconvenient in that it consumes large amounts of reagents per sample and produces noxious gases, which make harm to human health, accelerate wear out of laboratory equipment, and are unfriendly to the environment. Borates, on the other hand, are free of these disadvantages. Melting with lithium metaborate proceeds fast and efficiently, and the resulting product is easy to handle. This method has been in use since 1956 for the preparation of solid targets for the determination of chemical elements by x-ray fluorescence[4] and, since 1964, by spectrometric methods following dissolution of the melt in nitric acid[5,6,7]. Recently Sill and Bohrer[8] have employed fusion with $LiBO_2$ to prepare urine and faecal samples for the determination of uranium, plutonium, americium, thorium, and neptunium by alpha spectrometry.

No attempt has been made so far to apply this decomposition method to the analysis of $^{226}$Ra. When exploring this area we have discovered that, if the melt is brought in contact with a solution containing sulfate ions, a finely dispersed suspension of $BaSO_4$ is formed, which catches nearly all radium present in the sample. The size of the crystals is small enough to enable analysis by alpha spectrometry. We have also found that no thorium and uranium isotopes are captured by the $BaSO_4$ suspension, which enables their subsequent separation and determination from the filtrate.

This paper describes a detailed procedure for sequential determination of $^{226}$Ra and uranium isotopes in sediment and soil samples. Sources of $Ba(Ra)SO_4$ for alpha spectrometry can be prepared in less than 2 h in most cases. Only three simple operations are needed, namely fusion with $LiBO_2$, dissolution of the melt, and filtration. It is probably the fastest and the least laborious method available at present.

### EXPERIMENTAL METHODS

All reagents used were of analytical grade. Distilled water was used throughout. All solutions, with the exception of acids and organic solvents, were filtered through a membrane filter (pore size 0.2 µm) and stored either in polyethylene or in borosilicate glass bottles.

The glassware was new, not previously used for other purposes. After the use it was washed with a detergent solution and kept in a 1% ammonia solution of EDTA at pH 9–10.

#### Reagents

- Lithium metaborate: an anhydrous regent in granular form (0.3–0.5 mm) purchased from Claisse Corp., Sainte Foy, Quebec, Canada. This reagent can also be prepared by mixing $Li_2CO_3$ and $H_3BO_3$ taken in molar proportion 1:2[7,8].
- Sodium iodide or bromide: a solid substance.
- The solution for dissolution of the melts: dissolve 60 g of $Na_2SO_4$ and 29 g of citric acid in water, acidify the solution with 14 ml of concentrated sulfuric acid and dilute up to 1 l.
- 10% EDTA solution: dissolve 50 g of ethylenediaminetetraacetic acid disodium salt dihydrate in water containing 20 ml of concentrated ammonia (25%) and dilute up to 500 ml.
- Copper sulfate solution: dissolve 16.77 g of $CuSO_4{\cdot}5H_2O$ in water and dilute to 250 ml.
- $Ba^{2+}$ solution for the preparation of crystal seeds: dissolve 3.72 g of $BaCl_2{\cdot}2H_2O$ in water and dilute up to 50 ml.
- 70% $NaHSO_4$ solution for the preparation of crystal seeds: add 50 ml of concentrated sulfuric acid to 200 ml of water and dissolve 120 g of anhydrous $Na_2SO_4$ in it.
- Seeding suspension of the $BaSO_{4:}$ place 5 ml of a 70% $NaHSO_4$ solution and 0.2 ml of the $Ba^{2+}$ solution in a quartz or a Pyrex glass beaker, evaporate the water and heat the residue on a burner to obtain a clear melt. After cooling, dissolve the melt in a mixture of 12.5 ml of a saturated $Na_2SO_4$ solution and 12.5 ml of water. A volume of 0.1 ml of this suspension contains 50 mg of $BaSO_4$. A freshly prepared suspension can be used for approx. 2 weeks without losing its properties.
- Solution of the $^{133}$Ba tracer in 0.1 M $HNO_3$ containing 0.1 mg/ml of the stable $Ba^{2+}$ ions. The activity of the tracer should be established experimentally in such a way as to ensure the desired level of precision (e.g., ±1% RSD) within a reasonable counting period (e.g., 10 min).
- Standard reference solution of $^{226}$Ra: SRM 4967 (NIST) solution with initial activity concentration of 2729 Bq/g and combined standard uncertainty of 0.4%.
- pH indicator, preferably a 0.1% solution of bromocresol blue in 20% ethanol, or any other indicating pH variations within the pH range 3–5.
- Magnetic stirrer with a Teflon covered stirring bar.
- Platinum crucible of 10 or 20 ml capacity with smooth walls and a lid.
- Alpha spectrometer with solid-state detectors.
- Gamma-ray spectrometer.

#### *Additional Materials and Devices for Assaying Uranium*

- $^{232}U$ tracer: a solution of accurately known activity of the order of 1 Bq/ml.
- Dowex AG 1 × 4 (100–200 mesh) ion-exchange resin in the chloride form.
- Ion-exchange columns (10 × 150 mm) with funnels.
- Plating solution: dissolve 100 g of $(NH_4)_2SO_4$ in 900 ml of water, acidify with $H_2SO_4$ to pH 2.0 and dilute to 1 l.
- Other reagents: 10% $Na_2SO_4$ solution, 2 and 0.05 M sulfuric acid solutions, 60–70% perchloric acid, ammonia solution (conc.).
- Apparatus for electrodeposition consisting of Teflon plating cells, platinum electrodes, silver or stainless steel discs with a polished surface, D.C. power supply for a voltage of up to 20 V, and adjustable stabilized current of up to 1 A.

#### *Calibration Sources*

- Reference $^{133}Ba$ source for tracer recovery estimation: cut out a disk from a thick filtration paper. The diameter of the disk must be the same as the diameter of the active area of the $Ba(Ra)SO_4$ precipitate. Paste it on a suitable planchet. Apply known amount (weigh to the nearest 0.1 mg) of the $^{133}Ba$ tracer solution and let it dry. Cover the surface with a thin protective foil and count it together with the samples. Correct the count rate for the difference between the amount of tracer added to the samples and that present in the reference source.
- Reference source of $Ba(Ra)SO_4$ for detector efficiency calibration: in a small beaker place known amounts of $^{133}Ba$ tracer and $^{226}Ra$ standard solutions (weigh to the nearest 0.1 mg ). Add 0.1 mg of Ba carrier and dilute to 5 ml. Add 0.1 ml of the seeding suspension followed by 5 ml of saturated $Na_2SO_4$ solution. Collect the precipitate on a filter with the pore size of 0.1 µm. Determine the amounts of the radionuclides in the filtrate and on the walls of the glassware and make the respective corrections, as described in the procedure below.

### PROCEDURE

#### Determination of $^{226}Ra$

Place 0.1–0.5 g (preferably 0.2–0.3 g) of finely powdered ashed material in the Pt crucible and add the $^{133}Ba$ tracer. Both the sample and the tracer solution must be weighed to the nearest 0.1 mg to minimize weighing error. Bring the contents to dryness, avoiding spattering. Weigh in 1 to 1.5 g of $LiBO_2$ with the accuracy to the nearest 1 mg. The flux-to-sample ratio should be close to 5. Cover the crucible with a lid and heat it on a gas burner, using an oxidising flame, until a homogenous melt is obtained with no visible particles inside. Usually the dissolution takes 3–10 min, when intermittent swirling is applied. Refractory minerals, or coarser-grained material, may require a longer heating (30–40 min) before a complete sample decomposition is achieved.

Remove the crucible from the burner, cool it and add a pinch (0.05–0.1 g) of NaI or NaBr and resume heating until the contents turn to liquid. As soon as the melt assumes a ball-like shape, roll it gently around the crucible walls until all fine beads of molten material that may adhere to them combine with the bulk of the melt. Then quickly transfer the ball to the beaker containing 50 ml of the dissolving solution. The solution must be stirred vigorously with a Teflon stirring bar. Immediately add 50 ml of the seeding suspension and continue stirring until the melt has dissolved. Then add another 50 ml of the seeding suspension and continue mixing for another 5 min. Collect the precipitate by vacuum filtration on a membrane filter with pore size of 0.1 mm, wash the beaker and the filter chimney with water followed with 80% ethanol, and pass the washes through the filter.

Place the filter in a gamma spectrometer and acquire a sufficient number of counts to ensure good precision of the tracer recovery (1% or better). The recovery is expressed as a ratio of the count rate of the filter with $Ba(Ra)SO_4$ sample to the count rate of the reference filter which has the same active surface area as the sample filter and holds the same amount of $^{133}$Ba activity as that added initially to the sample.

The schematic diagram of the method is shown in Fig. 1.

If the calculated $^{133}$Ba recovery is unacceptably low, the following remedial steps can be applied:

– Using a disposable transfer pipette, rinse the filter chimney thoroughly with 5 ml of water containing 1 ml of 10% EDTA solution and 3 drops of conc. ammonia. Apply the washing solution several times to ensure complete removal of the $Ba(Ra)SO_4$ from the walls of the chimney. Complete the rinsing with a small volume of water.
– Transfer the washing solution to the beaker in which the melt was being dissolved, scrub the walls with a rubber policeman to detach and dissolve any adhering $Ba(Ra)SO_4$ precipitate, and add 0.1 ml of the $Ba^{2+}$ carrier solution (0.1 mg Ba).
– Check the $^{133}$Ba activity in the filtrate. If it is more than. e.g., 10% of the added tracer, combine it with the washing solution in the beaker; otherwise proceed to the next step.
– Add a few drops of pH indicator solution and adjust the pH to <5 with a few drops of acetic acid. Immediately add 0.1 ml of the seeding suspension followed by 5 ml of saturated $Na_2SO_4$ solution, and mix well.
– Introduce 1 ml of the $CuSO_4$ solution to bind into complex the excess of EDTA.
– Collect the $Ba(Ra)SO_4$ precipitate on a membrane filter and determine the $^{133}$Ba recovery by gamma counting.
– Retain the filtrate for uranium analysis, if such is required.

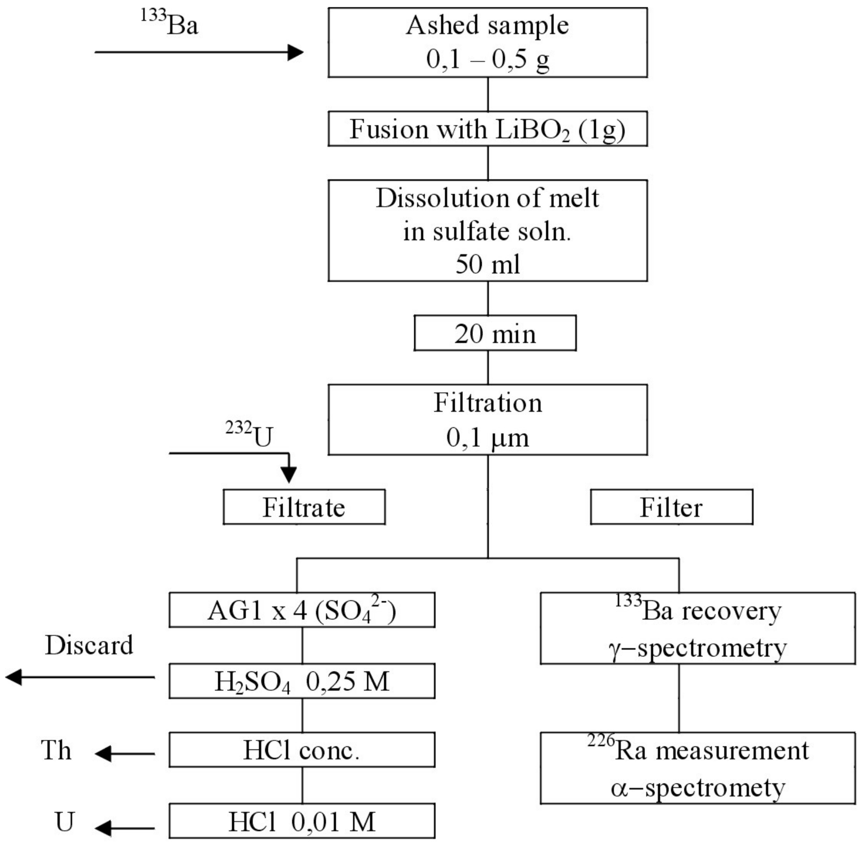

**FIGURE 1**. Analytical scheme for the determination of $^{226}$Ra and uranium isotopes.

To determine $^{226}$Ra activity on the filters, place them in the alpha spectrometer and acquire a sufficient number of counts in the main $^{226}$Ra peak to reach the desired statistical counting error.

### Determination of Uranium Isotopes

Uranium is determined in the filtrate after separation of Ba(Ra)$SO_4$ precipitate. Add an accurately measured amount of the $^{232}$U tracer and 3 g of $(NH_4)_2SO_4$ and mix well to dissolve the salt. Load the column with the Dowex AG 1×4 ion-exchange resin up to a height of 11 cm and convert it to a sulfate form by passing through it 50 ml of 2 M sulfuric acid followed by 20 ml of 0.05 M acid. Load the sample solution onto the column and let it run through by gravity. Wash the resin bed with 50 ml of 0.05 M sulfuric acid and then with 20 ml of concentrated hydrochloric acid. Reject the eluates. Uranium is eluted with 50 ml of distilled water.

Add 6 drops of a 10% $Na_2SO_4$ solution to the eluate and evaporate the solution to dryness. Treat the residue with concentrated nitric acid and heat until all organic matter is destroyed and the salts become white. Dissolve the residue with the plating solution and transfer the solution to the plating cell. To ensure good plating conditions the total volume of sample and washing solution should be kept small (7–10 ml) and the distance between the electrodes should be maintained at 5 mm. The current density should be adjusted at 0.5–0.8 A/cm$^2$.

After 2 h add to the plating cell a few drops of concentrated ammonia solution and stop the electrolysis. Rinse the disk with water and acetone and place it in the alpha spectrometer for counting.

Note: adding the $^{232}$U tracer to the sample after the Ba(Ra)$SO_4$ has been separated helps reduce interferences due to the $^{224}$Ra which is always present in appreciable amounts in the aged uranium spike solution.

## CALCULATIONS

### Radium-226

Since there are no suitable isotopic tracers for application in rapid methods, the correction for $^{226}$Ra losses incurred in the course of analysis is estimated from the recovery of $^{133}$Ba. Based on the results obtained on double-spiked samples, it was found that the recovery of $^{226}$Ra was never lower than the recovery of $^{133}$Ba. It is, therefore, safe to assume that it lies somewhere between the recovery of $^{133}$Ba and 100%. The midpoint of this range was chosen as an estimate of the $^{226}$Ra recovery, the range itself being a measure of the uncertainty on this estimate.

Obviously, the higher the $^{133}$Ba recovery, the smaller the uncertainty on $^{226}$Ra recovery. Therefore, an effort should be made to minimize analyte loss, e.g., by recovering it from the filtrate and the walls of the vessels.

For example, if the recovery of $^{133}$Ba tracer on the first and the second filter was 85 and 9%, respectively, and the respective activities of $^{226}$Ra were 90 and 5 mBq, the total $^{226}$Ra activity will be:

$$\frac{90}{\left(\frac{1+0.85+0.09}{2}\right)} = 98\,\text{mBq}$$

The activity calculated from the first filter only would yield:

$$\frac{90}{\left(\frac{1+0.85}{2}\right)} = 98\,\text{mBq}$$

### Uranium Isotopes

The activities are calculated using the well-known formula for isotope dilution method, e.g.:

$$A\ ^{238}U = A\ ^{232}U \frac{\sum \text{counts}\ ^{238}U}{\sum \text{counts}\ ^{232}U}$$

where $A^{232}U$ is the amount of $^{232}U$ activity, in Bq, introduced to the sample. The same applies to the remaining uranium isotopes.

### Blank

When the $^{226}$Ra content of the sample is expected to be low, the amount of radium introduced with the reagents should be determined and subtracted. The acids and sodium sulfate used in this work did not introduce detectable amounts of radium. The lithium borate was found to contain 0.23 ± 0.03 mBq $^{226}$Ra/g. Although a reagent of a better purity can be obtained by crystallization from an aqueous solution[7], the efficacy of that treatment for $^{226}$Ra has not been verified. Barium salts contain, as a rule, large and variable amounts of radium. That may pose problems when large amounts of barium are used as a coprecipitation agent. In this work, the amount of barium introduced with the $^{133}$Ba tracer, and the seeding suspension, was about 0.1 mg and the corresponding $^{226}$Ra contribution did not exceed 0.02 mBq per sample.

### Uncertainties

The uncertainty budget comprises six components: weighing error of samples (±0.1% standard uncertainty – SU) and $^{133}$Ba tracer (±0.2% SU), uncertainty of alpha particle detectors efficiency (±3% SU), uncertainty of $^{133}$Ba recovery in the Ba(Ra)$SO_4$ (±2% SU), uncertainty of $^{226}$Ra recovery, and uncertainty associated with alpha particles counting (statistical counting error). While the first four components can be kept under control, the remaining two vary from sample to sample and from one analysis to another. As described in the section “Calculations”, the uncertainty of $^{226}$Ra recovery results from the assumption that the latter lies somewhere between the recovery of $^{133}$Ba and 100%. Assuming that the best estimate is the midpoint between the two values, the maximum uncertainty will be expressed as a half of this interval. The numerical value obtained must be divided by √3 to express it in terms of SU[9]. Thus, using the values given in the section “Calculations”, the uncertainty of $^{226}$Ra recovery will amount to (100 – 94)/√3 = 3.5% when double Ba(Ra)$SO_4$ precipitation is made, or (100 – 85)/√3 = 8.7%, when only the first precipitate is considered.

The error on counting alpha particles is assumed to follow Poisson distribution, hence it is determined by the total number of net counts collected and the magnitude of the method blank, including detector background.

The total standard uncertainty is estimated as a square root of the sum of individual uncertainties taken to the power of two, in accordance with the law of propagation of uncertainties. Considering the unavoidable components of uncertainty arising from detector calibration and recovery estimations, it is reasonable to assume that under favourable conditions the total standard uncertainty cannot be much better than ±5%. However, it can be kept well below ±10% in most practical cases, i.e., when 0.2–0.3 g of material containing 30 Bq/kg or more of $^{226}$Ra is analysed, the recovery of Ba(Ra)$SO_4$ is not worse than 90% and the source is counted overnight on a detector with low background (<0.005 cpm) and 30% efficiency.

### Minimum Detectable Concentration

The lowest detectable concentration estimated from the Currie equation[10] for the measurement conditions given above is:

$$MDC = \frac{2.71 + 4.65\sqrt{Sb}}{\eta_{det.} \times R_{Ra} \times t \times M} = 0.34\ Ba/kg$$

where Sb is the standard uncertainty of blank (0.005 cpm), $R_{Ra}$ is the $^{226}$Ra recovery (0.95), t is time (1440 min), M. is the sample mass (0.3 g), and $ç_{det.}$ is the detector efficiency (0.3).

### RESULTS

The method was tested on a number of real samples with known activities of $^{226}$Ra. Among these were seven IAEA Reference Materials (RM) and two proficiency test samples issued by the Environmental Measurement Laboratory (EML) within the Quality Assessment Program (QAP). In Table 1 are shown the results obtained, together with the expected target values.

Figures 2A and 2B show a typical alpha spectrum obtained on the IAEA-306 sediment sample. The source was prepared by filtration of the Ba(Ra)$SO_4$ precipitate through a filter of 20-mm diameter and counted with a Si-PIPS detector of the same active area as the source, in a close distance (2

**TABLE 1**
**Results of $^{226}$Ra Measurements in Selected Reference Materials**

| | | | | | $^{226}$Ra Activity Bq/kg Confidence Interval (α = 0.05) | | |
|---|---|---|---|---|---|---|---|
| **No.** | **Material** | **Ref.*** | **Sample Code** | **Status**** | **Target Value** | **Our Value** | **Number of Samples Analysed** |
| 1 | North Sea sediment | [11] | SDN-1 | R | 109<br>106 – 120 | 107<br>100 – 114 | 4 |
| 2 | Baltic Sea sediment | [12] | IAEA-300 | I | 56.5<br>41.3 – 76 | 71<br>67 – 76 | 4 |
| 3 | Baltic Sea sediment | [13] | IAEA-306 | R | 66<br>60 – 81 | 110<br>95 – 124 | 4 |
| 4 | Irish Sea sediment | [14 ] | IAEA-135 | R | 23.9<br>20.6 – 25 | 33.5<br>31.1 – 35.9 | 4 |
| 5 | Black soil (Russia) | [15] | IAEA-326 | R | 32.6<br>31.0 – 34.2 | 34.6<br>31.5 – 37.7 | 4 |
| 6 | Soil (Russia) | [16] | IAEA-375 | R | 20.0<br>18.0–22.0 | 21.7<br>19.7 – 23.7 | 4 |
| 7 | Soil (Indonesia) | [17] | IAEA-312 | R | 269<br>250 – 287 | 293<br>281 – 305 | 17 |
| 8 | Soil (EML-USA) | [18] | QAP-49 | PT | 29 ± 1 (SD) | 32.8<br>28.4 – 37.2 | 4 |
| 9 | Soil (EML-USA) | [18] | QAP-53 | PT | 83.3 ± 4.9*** | 115<br>105 – 125 | 2 |

* Ref. – Reference.
** R – Recommended value, I – Information value, PT – Proficiency Test sample.
*** $^{214}$Bi value.

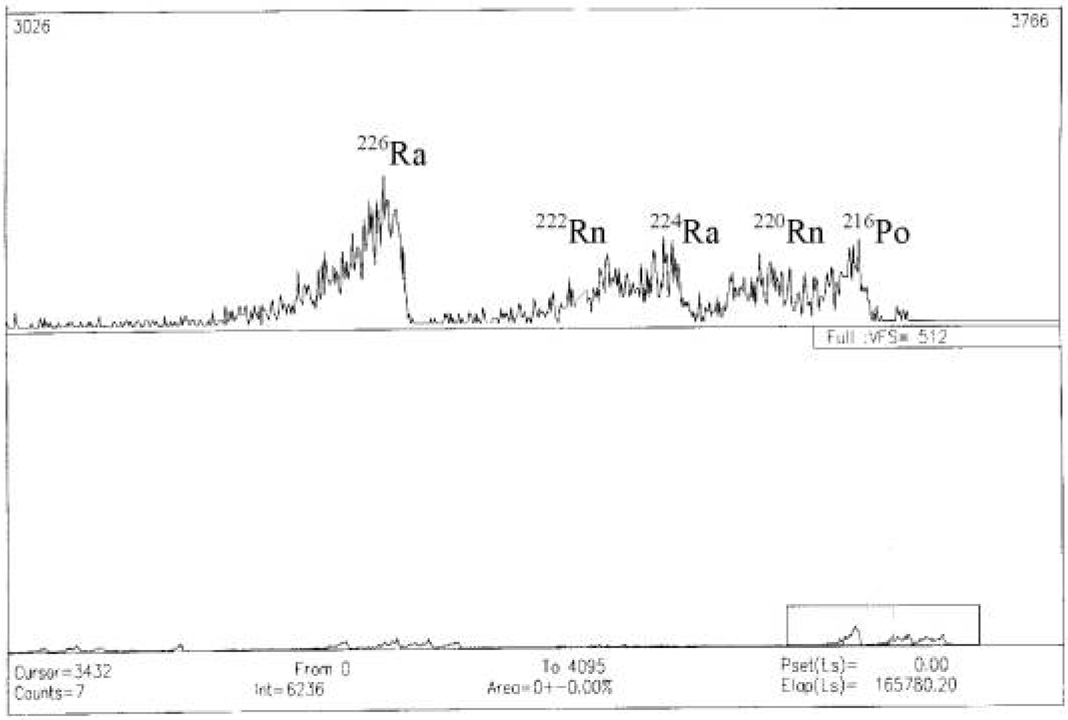

**FIGURE 2A**. Alpha ray spectrum for a sample IAEA-306 (Baltic sediment) measured right after source preparation.

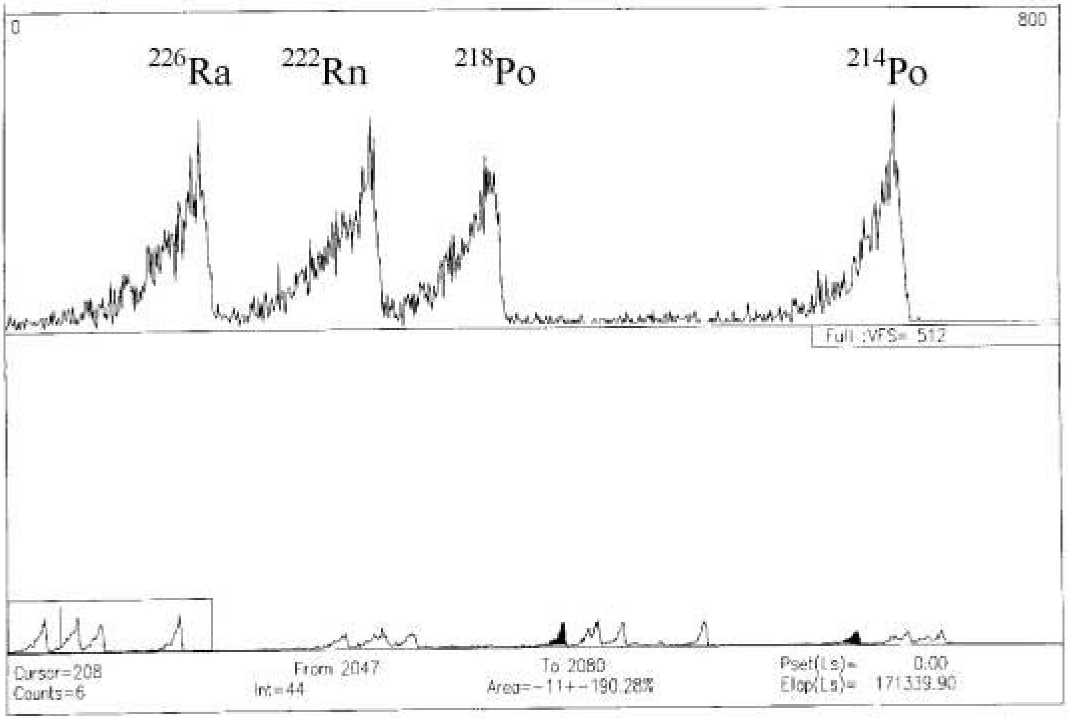

**FIGURE 2B**. Spectrum for the same sample measured a month later.

mm). The peaks, while broad, were separated well enough to enable integration of the counts resulting from the decay of $^{226}$Ra nuclei.

A comparison of the obtained results with the expected target values shows a fair agreement in most instances, but there are cases where the results differ significantly. The causes of the observed discrepancies are difficult to explain.

As can be seen, our method tends to yield somewhat higher values in general, compared to the target values. It seemed appropriate, therefore, to check whether the observed discrepancies are due to any $^{234}$U and $^{230}$Th atoms present in the Ba(Ra)$SO_4$ precipitate. The interference from $^{234}$U was examined by looking for the presence of the $^{238}$U peak in the 4.20 MeV energy region. No traces of such a peak were detected in any of the examined spectra. To determine a possible contribution of $^{230}$Th to the $^{226}$Ra peak, a reference thorium ore IAEA-RGTh-1 with the $^{232}$Th activity of 3,250 Bq/kg[19] was run through the described procedure. The spectrum recorded immediately after preparation of the Ba(Ra)$SO_4$ source is shown in Fig. 3A. Its shape indicates the absence of peaks corresponding to $^{232}$Th (4.01 MeV) and $^{228}$Th (5.42 MeV). The source was remeasured 3 weeks later, after which 98% of the initial $^{224}$Ra had decayed, making the $^{232}$Th energy region easier to examine (Fig. 3B). Again, no distinct $^{232}$Th peak has emerged in this region (the scale has been expanded tenfold compared to that in Fig. 3A). The contribution of $^{230}$Th would have been estimated at below 1%, had there been any. The spectrum of the "aged" source has revealed the presence of $^{226}$Ra, whose activity can be estimated at approx. 3% of the $^{232}$Th activity present in the sample.

It follows from the foregoing that spectral interferences are unlikely to account for the differences between the obtained results and the target values. Inasmuch as the sources of uncertainties are few, and all of them are quantifiable, there is no reason to believe that the method is seriously biased. However, it should be borne in mind that the materials on which the method was tested were not of the highest metrological quality. It cannot be excluded, therefore, that some of the reference

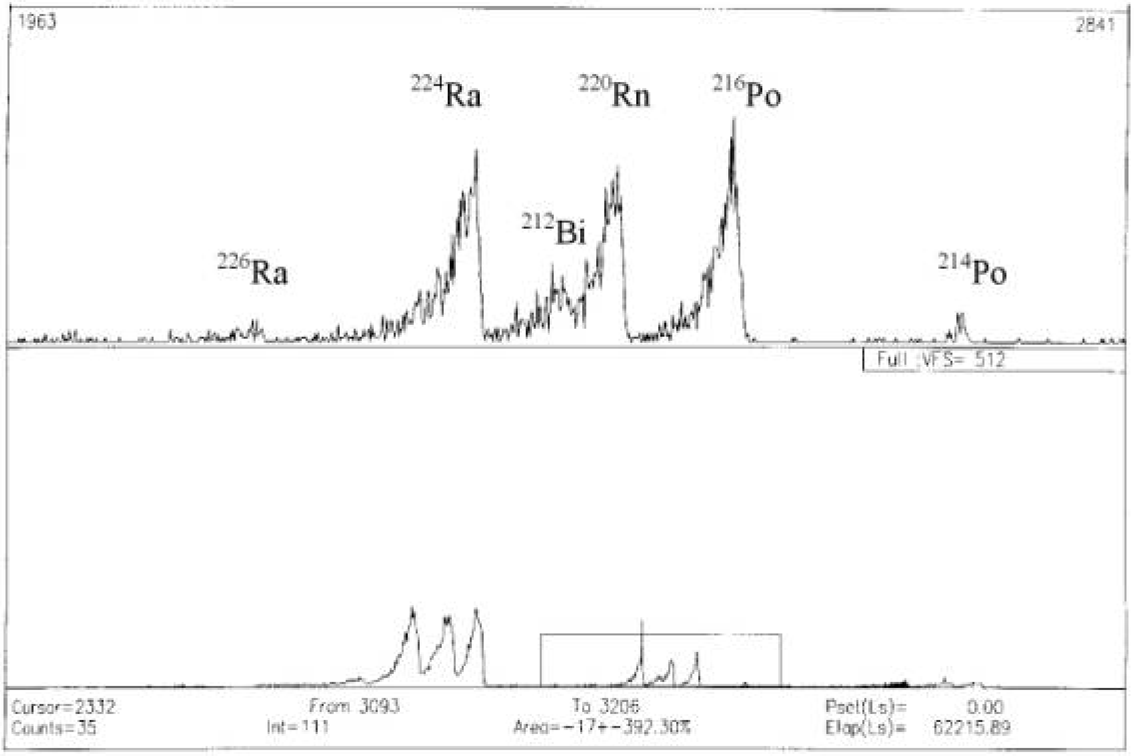

**FIGURE 3A**. Alpha ray spectrum for a sample IAEA RGTh-1 (thorium ore) measured right after source preparation.

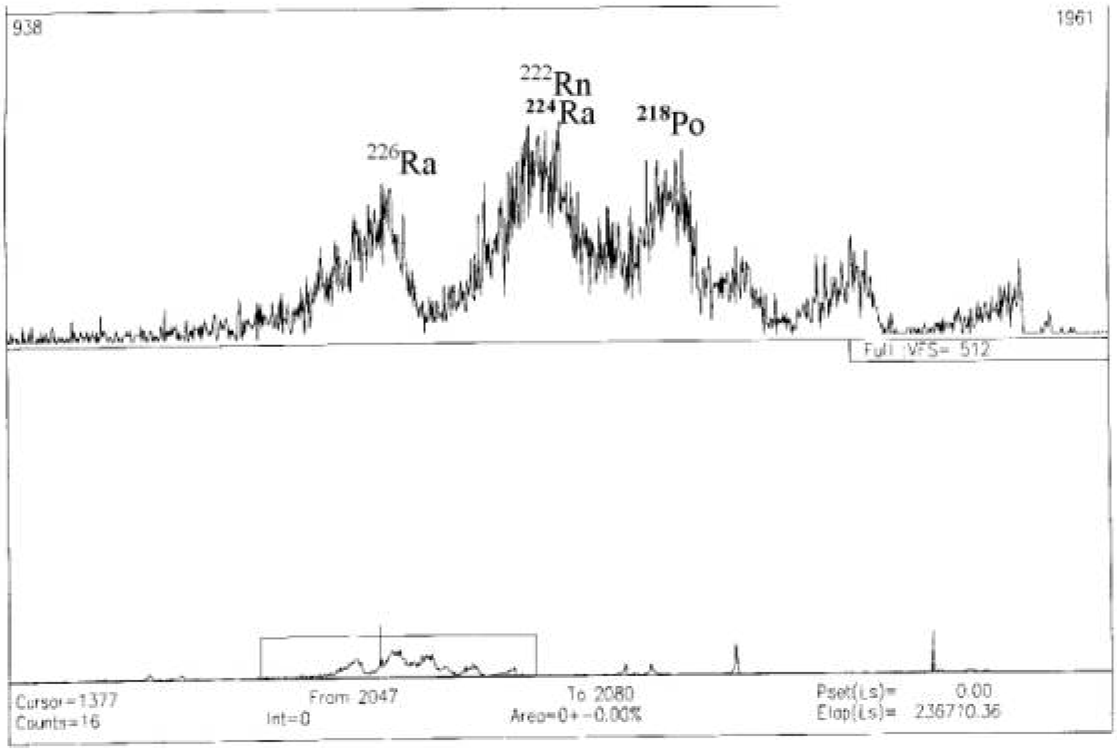

**FIGURE 3B**. Spectrum for the same sample measured 3 weeks after source preparation.

values of the materials used in this comparison are biased due to deficiencies inherent in the methods by which they have been established.

As long as natural matrix reference materials with accurately established reference values are unavailable, this dilemma will remain unresolved.

### DISCUSSION

Unlike most of the available radioanalytical procedures for $^{226}$Ra, this method is simple, fast, and easy to employ in a modestly equipped laboratory with access to alpha- and gamma-ray spectrometers. It requires neither expensive reagents nor skilled personnel. Several precautions should, however, be taken to avoid some pitfalls.

#### Sample Size and Flux-to-Sample Ratio

Several factors play a role in selection of the optimum sample size. As a rule, the smaller the sample, the faster the decomposition and dissolution of the melt is. On the other hand, with larger samples better count rate is achieved, hence better precision is arrived at within the same period of time. The upper limit on the sample size is set by the amount of the stable barium present in the analysed material. Concentrations of $Ba^{2+}$ in most soils and sediments fall within the range of 50–1,000 mg/g. Experience shows that the amount of $Ba(Ra)SO_4$ on the filter should be kept below 200 mg/cm$^2$ if acceptable spectral resolution is to be obtained. A filter with an active filtration area of 3.14 cm$^2$ (ø 20 mm) can thus be loaded with up to 370 mg of $Ba^{2+}$. If we subtract the amount of $Ba^{2+}$ introduced to a sample with $^{133}$Ba tracer and seeding suspension, we arrive at 0.3 g as a safe upper limit for sample size with respect of its $Ba^{2+}$ content.

Another limitation is imposed by solubility of the melts. To ensure the highest $Ba(Ra)SO_4$ recovery, the volume of solution should be kept as small as possible. It was found experimentally, that 50 ml is the minimum volume, in which a melt of 0.3 g soil/sediment with 1.5 g $LiBO_2$ can be dissolved in a reasonable period of time (20–60 min).

The flux-to-samples ratio depends somewhat on the nature of sample. Some sediments can be totally molten at a 2:1 wt. ratio, but most of the soil samples tested form well-behaved melts at a 5:1 ratio. The sample undergoes complete decomposition within 5–10 min and the melt dissolves completely in less than 1 h.

### Melting and Dissolution of the Melt

Melting proceeds rapidly when the material is finely ground and thoroughly mixed with $LiBO_2$. Intermittent swirling of the melt in the crucible speeds up dissolution of the material in the flux.

The material should be free of reducing substances because they may damage the surface of Pt crucibles. Any Pt that passes into the melt will eventually co-precipitate with $Ba(Ra)SO_4$ and deteriorate resolution of the peaks in the alpha spectra. Organic matter and sulfides should be oxidized by roasting the material at 700 K. An oxidizing flame should be used throughout the melting process.

Once a homogeneous melt is obtained, it is poured into a glass beaker containing 50 ml of the dissolving solution. Addition of sodium iodide or bromide makes the walls of Pt crucible unwettable and ensures complete removal of the melt. The walls must be kept shiny to prevent small droplets of the melt from sticking to the walls. A 95%Pt+5%Au alloy is recommended as material for making crucibles[20]. Alternatively, graphite crucibles (“vitreous carbon”) may be used[7]. They have advantage of being intrinsically unwettable, thus eliminating the need of using iodides or bromides. However, melting must be carried out in a muffle furnace rather than on a gas burner.

The temperature of melt at the moment of pouring out is important. Claisse[20] recommends to adjust it to about 1,100 to 1,200 K. At this temperature the ball of melt shatters to form fine flakes when it comes in contact with the liquid. When the melt is too hot, it does not fall apart into small pieces and the dissolution may take hours. Also important is the initial temperature of the receiving solution. The optimum range is 295–303 K. When the temperature is beyond this range, the dissolution becomes very slow. Attention must be paid to stir the solution vigorously, especially at the moment of pouring the melt into the beaker. Under such conditions the operation is safe and proceeds without splashing of the liquid. Care should be taken to transfer the melt completely in one step. If a part of the melt remains in the crucible and is added to the solution when cold, it will remain undissolved for hours.

### Filtration

Millipore or Sartorius vacuum filtration units with effective filtration diameter of 20 mm match well the most popular detectors with 300 or 450 $mm^2$ of active surface. Filtration takes 6–7 min. Sill[3] gives useful hints for the preparation of the filtration apparatus and for conducting the filtration in such a way as to minimize adsorption of precipitate on the walls during filtration. In order to ensure good retention of the $Ba(Ra)SO_4$ precipitate on the filter, it is recommended to use filters with pore size of 0.1 mm. The glassware which is in contact with the sample suspension should be unscratched, preferably new, and not used for other purposes. The walls should be kept wettable. The chimney of the filtration apparatus should be wet throughout the filtration. The beaker in which the melt was being dissolved, and the filtration chimney, should be rinsed with water, followed by 80% ethanol. Immediately after filtration, the filter should be gamma-counted to determine the $^{133}Ba$ recovery. If it is below 90% or, if one strives for a high accuracy, an attempt should be made to recover the missing part of the radionuclides from the filtrate, and from the walls of the beaker and the filter chimney. The filtrate should be checked for the presence of the $^{133}$Ba first. If the activity of the latter does not exceed 10% of the added amount, it is not worthwhile recovering it, because the amount of the $^{226}$Ra lost to the filtrate is even smaller. The missing $^{226}$Ra activity is to be found on the walls of the beaker and the chimney and can be recovered as described in the procedure.

The partition of $^{133}$Ba among the filter, the filtrate and the walls of the beaker and chimney was determined in the samples listed in Table 1. A summary of the results is given below:

| Item | $^{133}$Ba (%) On the Filter | In the Filtrate | On the Walls |
|---|---|---|---|
| Mean recovery ± $1s_{n-1}$ | 90.8 ± 4.7 | 4.4 ± 2.2 | 4.5 ± 4.4 |
| Range | 76.4 – 98.0 | 1.6 – 8.9 | 0.3 – 14 |
| Number of samples | 27 | 27 | 27 |

It is evident, that little $^{133}$Ba, and still smaller amounts of $^{226}$Ra, are lost to the filtrate. However, the amounts of radionuclides remaining on the walls may be sometimes large enough to justify their recovery.

### Other Considerations

The method fails to perform well in the presence of large amounts of the calcium and strontium ions. When the concentration of calcium in the analysed material exceeds 30%, the calcium ions liberated from the melt combine with sulfate ions present in the dissolving solution to form insoluble calcium sulfate. Since the process of calcium sulfate formation is slow, the filtration can be sometimes completed before the precipitate appears. Once formed, it can be redissolved by addition of more water, but the recovery drops markedly and one or two additional precipitations must be carried out to ensure a better than 90% recovery. In such cases it is advisable to repeat the analysis using less material and extending the counting time.

The formation of $Ba(Ra)SO_4$ in the dissolving solution was found to depend on the kind of cation in the sulfate salt used for its preparation. The highest recoveries were noted with lithium sulfate. Sodium and ammonium salts worked nearly as well as the lithium salt, but in solutions of potassium or rubidium sulfates practically no precipitation was formed. These ions should, therefore, be avoided.

Sometimes, the quality of the source is poor and the spectrum is difficult, or impossible to resolve. In such cases it is recommended to repeat the analysis using smaller amount of the material. One can also consider one of the following remedial procedures, if the material is in shortage:

– Fusion with sodium pyrosulfate: destroy the filter by wet or dry ashing, depending on the kind of the filter material. Add 1 ml of 70% $NaHSO_4$ and a few drops of $HClO_4$, and heat until a transparent melt is obtained. Cool and dissolve the melt in a mixture of 5 ml of the saturated $Na_2SO_4$ solution and 5 ml of distilled water. Proceed with filtration as described in the procedure.
– Fusion with $LiBO_2$: ash the filter in a ceramic crucible, transfer the ash to a Pt crucible, and mix with 0.5 g of $LiBO_2$. Heat shortly until a homogeneous transparent melt is obtained. Pour the melt into a 50-ml narrow beaker to which 20 ml of the dissolving solution was added and mix vigorously with a magnetic stirrer. Do not add seeding suspension until the melt is completely dissolved. If at that stage the solution is transparent, or only slightly opalescent, add 50 mg of $Ba^{2+}$ and 0.1 ml of the seeding suspension, continue mixing for another 10 min, and proceed with filtration.
– Dissolution in alkaline EDTA and reprecipitation: place the filter in a small beaker, add 1 ml of 10% EDTA solution and 3 drops of conc. ammonia solution, and dilute with water to cover the filter. Keep the beaker in an ultrasonic bath for 15 min and filter the solution through a 0.45-µm membrane filter and wash it with a small volume of water. Check the activity of $^{133}$Ba on the membrane filter and discard it if it is negligible. Transfer the filtrate to a beaker, add one drop of the pH indicator solution, and adjust the pH with acetic acid to 4–5. Immediately add 0.05 ml of the seeding suspension followed by 5 ml of saturated $Na_2SO_4$ solution and 1 ml of the copper sulfate solution. Collect the suspension on the same filter from which the Ba(Ra)$SO_4$ precipitate was removed.

The latest procedure has advantages over the fusion methods and is to be recommended.

### CONCLUSIONS

The described procedure is aimed at the analysts that are in need of a rapid and simple, yet accurate and sensitive method for $^{226}$Ra determination in mineral matrices. The method requires little workload and skills, and the cost per sample is small. It is particularly suitable in emergency situations and in cases where the amount of available material is limited (air dust, suspended particular matter, grains of minerals, thin layers of deposits, small intrusions, etc.). The method is probably the least laborious and the fastest available at present.

When time is not a critical factor, an isotopic tracer $^{225}$Ra can be applied to reduce the uncertainty in recovery determination. Work towards achieving this objective is in progress.
